# Acclimation to Nutritional Immunity and Metal Intoxication Requires Zinc, Manganese, and Copper Homeostasis in the Pathogenic *Neisseriae*

**DOI:** 10.3389/fcimb.2022.909888

**Published:** 2022-06-30

**Authors:** Alexis Hope Branch, Julie L. Stoudenmire, Kate L. Seib, Cynthia Nau Cornelissen

**Affiliations:** ^1^Center for Translational Immunology, Institute for Biomedical Sciences, Georgia State University, Atlanta, GA, United States; ^2^Institute for Glycomics, Griffith University, Gold Coast, QLD, Australia

**Keywords:** metal intoxication, nutritional immunity, *Neisseria gonorrhoeae*, *Neisseria meningitidis*, zinc, manganese, copper, integrated metal homeostasis

## Abstract

*Neisseria gonorrhoeae* and *Neisseria meningitidis* are human-specific pathogens in the Neisseriaceae family that can cause devastating diseases. Although both species inhabit mucosal surfaces, they cause dramatically different diseases. Despite this, they have evolved similar mechanisms to survive and thrive in a metal-restricted host. The human host restricts, or overloads, the bacterial metal nutrient supply within host cell niches to limit pathogenesis and disease progression. Thus, the pathogenic *Neisseria* require appropriate metal homeostasis mechanisms to acclimate to such a hostile and ever-changing host environment. This review discusses the mechanisms by which the host allocates and alters zinc, manganese, and copper levels and the ability of the pathogenic *Neisseria* to sense and respond to such alterations. This review will also discuss integrated metal homeostasis in *N. gonorrhoeae* and the significance of investigating metal interplay.

## Introduction - Pathogenic *Neisseriae* Cause Devastating Yet Distinct Diseases to the Human Host

*Neisseria gonorrhoeae* and *Neisseria meningitidis* are human-specific pathogens of significant public health concern. Despite high DNA and amino acid sequence identity, *N. gonorrhoeae* and *N. meningitidis* cause significantly different diseases (Tinsley and Nassif, 1996; Perrin et al., 2002).

*N. gonorrhoeae* is the causative agent of the second most common reportable infectious disease in the United States, gonorrhea, and predominantly colonizes the genital mucosal epithelium and oropharynx (CDC, 2019b). Symptomatic gonococcal infection presents as urethritis, cervicitis, salpingitis, pharyngitis or conjunctivitis (CDC, 2019b). However, gonorrhea can present asymptomatically as well. Asymptomatic infection in women is of great concern as it enables the pathogen to ascend to the upper reproductive tract, where it can cause pelvic inflammatory disease. Pelvic inflammatory disease can lead to ectopic pregnancy, scarring, infertility, and chronic pelvic pain. In 2018, the incidence of gonococcal disease was 179.1 cases per 100,000, correlating with a total of 583,404 reported cases in the United States (CDC, 2019b).

*N. meningitidis* can inhabit the nasopharynx without eliciting symptoms; this carrier state can be found in 5-10% of the US population (CDC, 2019c). The carrier state can transition to symptomatic disseminated infection, sometimes referred to as invasive meningococcal disease (IMD), which is characterized by nausea, vomiting, rash, stiffness of neck, fever, and diarrhea (CDC, 2019c). While the incidence of meningococcal disease has decreased dramatically from ~1.50 per 100,000 in 1980 to ~0.2 per 100,000 in 2018, IMD remains a severe threat to infants. Incidence of IMD in children younger than 1 year has averaged around 1.20 per 100,000 in the past 10 years (CDC, 2019a). About 12% of infections result in death, and some survivors experience permanent brain damage, loss of limbs or hearing loss (Candrilli, 2019; CDC, 2019a).

While the pathogenic *Neisseriae* pose a direct threat to human health, they also represent a substantial economic burden in the United States. In 2018, gonorrhea infections resulted in an estimated $85 million in direct medical costs in the United States (Kumar et al., 2021). The estimated total cost of the response to an IMD outbreak at the University of Oregon Hospital (7 cases) and the Oregon State University Hospitals (6 cases) was $12.3 million (Candrilli, 2019).

During neisserial infection, the host limits bacterial proliferation by modulating metal availability through two related mechanisms: nutritional immunity and metal intoxication. Nutritional immunity is characterized by host sequestration of free metals from the bacterial nutrient supply, limiting metals required for enzymatic and metabolic functions (Hennigar and McClung, 2016). Metal intoxication is the process by which the host overloads pathogens with toxic metal concentrations (Becker and Skaar, 2014). Metal overload in bacteria contributes to reactive oxygen species (ROS) and reactive nitrogen species (RNS) cycling (Djoko et al., 2012), protein mismetallation (Veyrier et al., 2011), and subsequent stalling of respiration (Djoko and McEwan, 2013). Pathogenic bacteria have evolved mechanisms to access restricted metals as well as limit metal overload.

Metal availability within a biological niche dictates pathogen survival and the extent of pathogenesis. Although causing distinct disease presentations, pathogenic *Neisseria* share many mechanisms of responding to metal scarcity and overload to ensure survival and maintain virulence. This review aims to provide an overview of the neisserial response to nutritional immunity and metal intoxication with respect to zinc, manganese, and copper.

## Zinc, Manganese, and Copper Are Allocated to Specific Host Niches

Transition metals, such as zinc, manganese, and copper, are essential to many host processes, including oxidative stress resistance (Girotto et al., 2014; Jarosz et al., 2017; Ganini et al., 2018), cell signaling and metabolism (Maares et al., 2018), immune modulation (Hu Frisk et al., 2017), post-translational modifications (Braiterman et al., 2015; Tidball et al., 2015), and structural maintenance and enzymatic processing (Tidball et al., 2015; Ganini et al., 2018). These metals play similar roles in pathogens including *Neisseria meningitidis* (Persson et al., 2001; Pawlik et al., 2012; Hecel et al., 2018) and *Escherichia coli* (Kaur et al., 2017*)*.

Zinc within the human body is primarily localized to the bone and skeletal muscle, with moderate concentrations found in the kidneys and liver (Jackson, 1989). Most zinc, however, is metabolically unavailable to the host due to slow zinc turnover with the exception being zinc found in the sperm (Baer and King, 1984). High levels of zinc-binding metallothioneins (Suzuki et al., 1994), which maintain zinc and copper homeostasis and limit heavy toxicity in host cells (Rahman and Karim, 2018), can be found in male secretions (Suzuki et al., 1992). It is feasible that zinc-binding metallothioneins help create a zinc limited environment for the gonococcus during male urethral infection. The majority of zinc within the host is not easily accessible to pathogens due to a limited pool of labile zinc (Brown et al., 2001), which can be further restricted during infection by production of calprotectin and other S100 proteins that act to sequester free zinc away from the pathogen (Yadav et al., 2020). Calprotectin makes up 45% of the protein content in neutrophils (Edgeworth et al., 1991) and is released following neutrophil death (Voganatsi et al., 2001) and Neutrophil Extracellular Trap (NET) formation (Urban et al., 2009). The zinc sequestering protein, S100A7, is enriched in lower genital tract epithelial cells (Mildner et al., 2010). In the case of mucosal infection by *N. gonorrhoeae*, a robust Th17 response results in the influx of neutrophils (Liu and Russell, 2011). Thus, S100A7 and calprotectin, which has been released by neutrophils, create a zinc limited environment for *N. gonorrhoeae* (Zackular et al., 2015). The remaining zinc is dispersed among the reproductive (Baer and King, 1984) and immune systems (Brown et al., 2001).

Within the human host, manganese exists as Mn^2+^ and Mn^3+^ (O'Neal and Zheng, 2015). Mn^2+^ is found in the blood bound to albumin, ß-globulin, bicarbonate, and citrate, and in the cytosolic content of neutrophils bound to calprotectin (O'Neal and Zheng, 2015; Zackular et al., 2015). Within the cell, Mn^2+^ is found at the highest concentrations in the endoplasmic reticulum and mitochondria, where it plays an antioxidant role through Mn-dependent superoxide dismutase (MnSOD) (Maynard and Cotzias, 1955; Ganini et al., 2018). In neurons, Mn^2+^ is required for signal transduction and enzymatic function (Gunter et al., 2013; Tidball et al., 2015). Excess Mn^2+^ accumulates in the liver, kidneys, bone, and pancreas, with higher levels bound to regulatory proteins in the brain and cerebrospinal fluid. Mn^3+^ can be bound to transferrin, which transports Mn^3+^ to neuronal cells in a mechanism similar to Fe^3+^ transport (Chen et al., 2001; Gunter et al., 2013). Many Gram-negative pathogens utilize Mn^2+^ (i.e. *Acinetobacter baumanii, Salmonella enterica, E. coli, Helicobacter pylori*, and *N. gonorrhoeae*), in the face of nutritional immunity, for oxidative stress resistance and metabolism (Tseng et al., 2001; Kehres et al., 2002b; Anjem et al., 2009; Lee et al., 2010; Diaz-Ochoa et al., 2016; Juttukonda et al., 2016). During gonococcal infection of macrophages, the manganese transport protein Natural resistance-associated macrophage protein 1 (NRAMP1) (Jabado et al., 2000), is upregulated on the phagosomal membrane (Forbes and Gros, 2003; Zughaier et al., 2014). NRAMP1 shuttles manganese from the phagosomal compartment to the cell cytosol to restrict pathogen access to manganese. Consequently *N. gonorrhoeae* may experience manganese limitation in the endolysosomal compartment following phagocytosis by macrophages (Ivanov et al., 2022).

Copper is found primarily in the liver and plasma in free and ceruloplasmin-bound forms (Dunn et al., 1991). Ceruloplasmin facilitates copper transport through the vasculature and possesses the ferroxidase activity necessary for the oxidation of Fe^2+^ to Fe^3+^ and subsequent iron loading of transferrin (Ramos et al., 2016; Hyre et al., 2017). Ceruloplasmin is also transported to the urinary tract during human infection (Hyre et al., 2017). Thus, *N. gonorrhoeae* may experience ceruloplasmin-dependent copper limitation in addition to S100 protein-dependent zinc limitation at the mucosal surface. Copper can also be found within the cytosol of neutrophils, which are recruited to the gonococcal infection site (Johnson and Criss, 2011; Sintsova et al., 2014), following import by CTR1 on the neutrophil membrane (Zhou and Gitschier, 1997; Cichon et al., 2020).

## Metal Acquisition by the Pathogenic *Neisseriae* in Metal-Restricted Niches Requires Highly Specific Metal Import

Metal import poses a particular challenge to Gram-negative bacteria, as it requires transport across a two-component cell wall. Outer membrane transport utilizes the proton motive force and requires energy transduction, *via* the Ton system, from the cytoplasmic membrane. Scarce metals (i.e., zinc, manganese, and copper) are transported into the cytoplasm in an ATP-driven mechanism, which is often tightly regulated to avoid metal overload, protein mismetallation, and oxidative stress. Highly specific metal transport is required in a host that uses metal sequestration to restrict microbial growth and pathogenesis.

**Figure 1:**
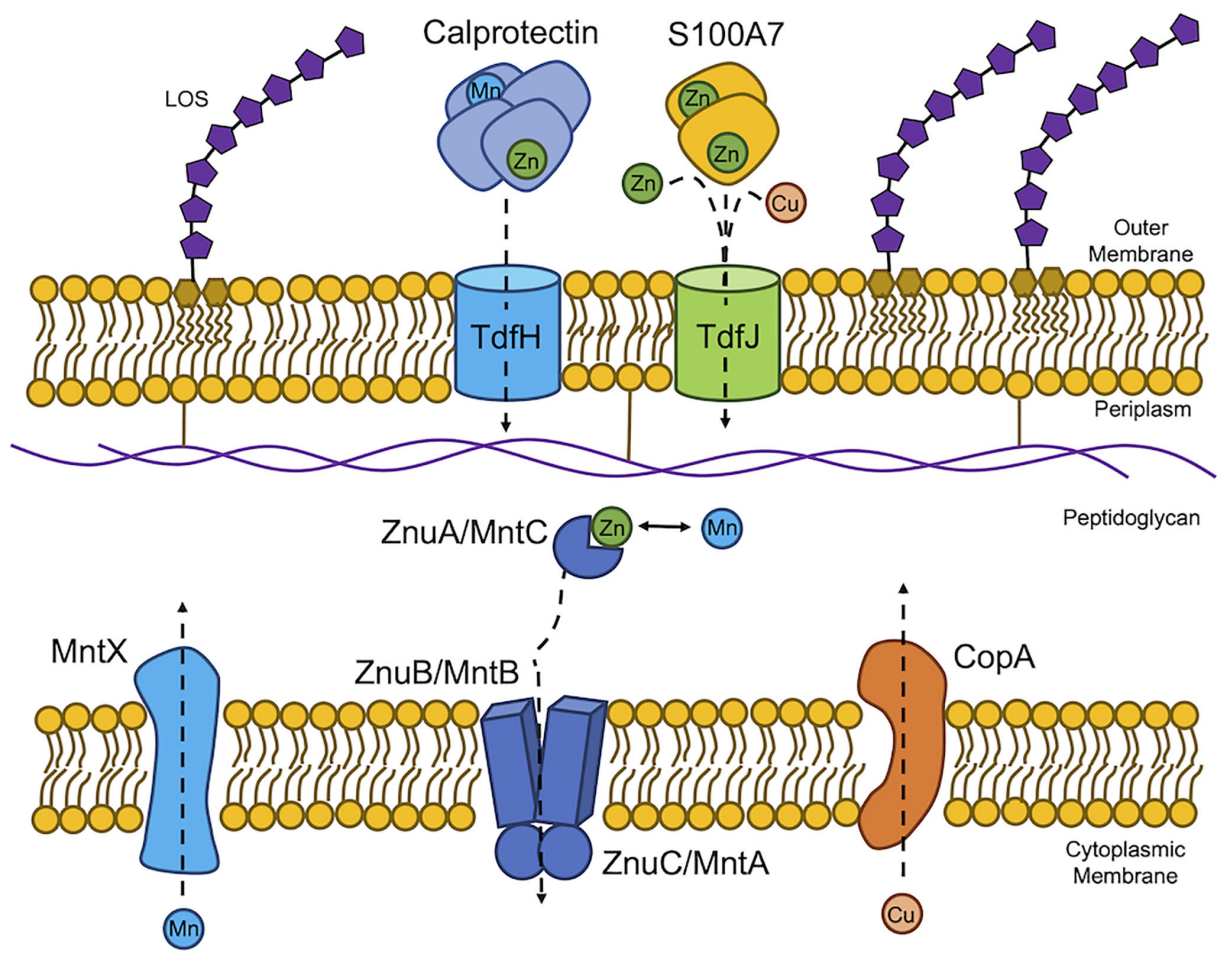
*Neisseria gonorrhoeae* and *Neisseria meningitidis* experience host-driven nutritional immunity and metal intoxication. The host exerts nutritional immunity on the pathogenic *Neisseriae* by exposing the bacteria to calprotectin and S100A7. Calprotectin sequesters both zinc (Zn) and manganese (Mn) from the extracellular environment to limit Zn and Mn availability. *N. gonorrhoeae* in turn expresses TdfH, which has been shown to strip Zn from calprotectin and subsequently import the ion. TdfH is able bind Mn-loaded calprotectin suggesting a role for Mn import across the outer membrane. TdfH is referred to as CbpA in *N. meningitidis.* S100A7 also sequesters Zn from the extracellular environment. Gonococcal TdfJ binds S100A7 to pirate and import the Zn payload. Lipooligosaccharide (LOS) is shown in the outer membrane for reference. Once in the periplasm, Zn and Mn are chaperoned by the periplasmic binding protein, ZnuA (MntC) to the permease in the cytoplasmic membrane, ZnuB (MntB). Transport across the cytoplasmic membrane is energized by the ATPase, ZnuC (MntA). The host also exerts metal intoxication, specifically copper (Cu) intoxication on *N. gonorrhoeae* and Mn intoxication in *N. meningitidis* through unknown mechanisms. In response, these efflux proteins export excess cytoplasmic Cu or Mn from to the periplasmic space.

In the human host, which allocates metals to specific niches, the pathogenic *Neisseria, N. gonorrhoeae and N. meningitidis*, have evolved mechanisms to acquire zinc, manganese, and copper in specific environments. Gonococcal TdfH and TdfJ are zinc-specific TonB-dependent transporters that pirate zinc from calprotectin (Kammerman et al., 2020) and S100A7 (Maurakis et al., 2019), respectively, to transport that zinc across the outer membrane to the periplasm (**Figure 1**). Gonococcal TdfH binds calprotectin through a high-affinity bimodal interaction. TdfH interacts with a tetramer of calprotectin, which itself is a heterodimer of S100A8 and S100A9 (Bera et al., 2022). Interestingly, gonococcal growth when calprotectin is the sole zinc source requires the presence of zinc in site 1 of calprotectin, the preferred zinc utilization site by gonococcal TdfH. Mutant calprotectin unable to bind zinc at site 2 fully supports gonococcal growth (Kammerman et al., 2020). The cryoEM structure of the calprotectin:TdfH complex has been determined by Bera et al. (Bera et al., 2022). Since site 1 of calprotectin is capable of binding both zinc and manganese (Gagnon et al., 2015) and gonococcal TdfH is able to bind manganese-loaded calprotectin (Bera et al., 2022), it is feasible that TdfH may also be a manganese importer. The meningococcal TdfH homolog was renamed calprotectin-binding protein A (CbpA); this protein has been shown to bind to zinc- or manganese-loaded calprotectin (**Table 1**) with higher affinity than it does to unloaded calprotectin, demonstrating a preference for metalated calprotectin over the apo form (**Figure 1**) (Stork et al., 2013). Gonococcal TdfJ was shown to bind S100A7 with high specificity and to use the human protein as a zinc source (Maurakis et al., 2019). Similarly, the meningococcal homolog of TdfJ is a zinc-specific importer required for zinc import during zinc limitation (Stork et al., 2010).

**Table 1:** *Neisseria gonorrhoeae* (Ng) and *Neisseria meningitidis* (Nm) express proteins which are potentially involved in integrated metal homeostasis.

Ng protein (Affinity [ligand])	Nm protein	Metals associated with ligand	Reference
TdfH (4 nM and 35 µM [calprotectin])	CbpA	Zn, Mn	(Pawlik et al., 2012; Stork et al., 2013; Jean et al., 2016; Kammerman et al., 2020)
TdfJ (nk, (S100A7))	ZnuD	Zn, Cu, Fe, Cd	(Jean et al., 2016; Hecel et al., 2019; Maurakis et al., 2019)
TbpB (7.4 nM [transferrin])	TbpB	Mn, Fe	(Cornelissen and Sparling, 1996; Ronpirin et al., 2001; Wu et al., 2010)
ZnuCBA/MntABC (100 ± 8 nM [Mn^2+^]; 104 ± 5 nM [Zn^2+^])	ZnuCBA/MntABC	Zn, Mn	(Chen and Morse, 2001; Tseng et al., 2001; Wu et al., 2006)
MntX (nk, [Mn^2+^])	MntX	Mn, Fe	(Veyrier et al., 2011)
Zur/PerR (nk, [Mn^2+^])	Zur	Zn, Mn	(Wu et al., 2006; Pawlik et al., 2012; Jean et al., 2016)

nk, (not known) indicates the affinity for that ligand is not known. These proteins have been shown to be regulated by or interact with multiple metals.

After crossing the outer membrane, metals must be escorted across the periplasm to transporters in the cytoplasmic membrane. Metal chaperoning across the periplasm is often accomplished by periplasmic metal-binding proteins (PBP) of the Cluster A-I substrate-binding protein family. PBP transport precedes the ATP-dependent transport step through the cytoplasmic membrane (Dintilhac and Claverys, 1997; Berntsson et al., 2010). Precise metal transport across the periplasm is required for acclimation to the specific metal environments encountered by Gram-negative pathogens (Lewis et al., 1999; Ammendola et al., 2007; Davies and Walker, 2007; Lim et al., 2008; Davis et al., 2009; Desrosiers et al., 2010; Pederick et al., 2015). PBPs deliver specific metals to permeases in the cytoplasmic membrane, where an ATPase then hydrolyzes ATP to energize metal transport into the cytoplasm.

**Figure 2:**
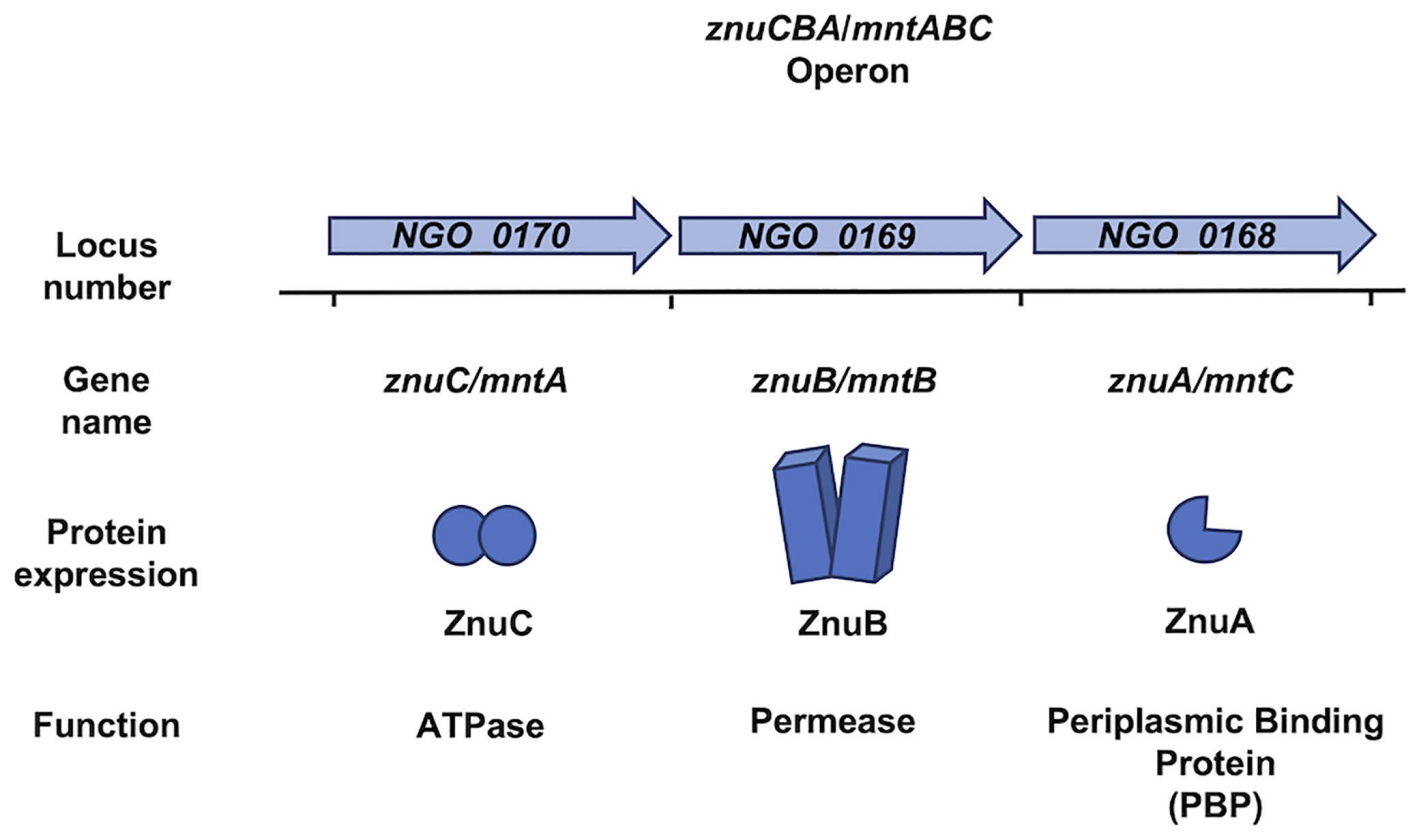
The genome of *Neisseria gonorrhoeae* and *Neisseria meningitidis* encodes the *znuCBA/mntABC* operon. The *znuCBA* operon in *N. gonorrhoeae* is indicated by the locus numbers NGO_0170, NGO_0169, and NGO_0168. The operon is named in order of predicted transcriptional direction. NGO_0170 encodes an ATPase named ZnuC which provides energy from zinc (Zn) transport into the cytoplasm. NGO_0169 encodes a permease named ZnuB which serves as a channel for transporting Zn through the cytoplasmic membrane. NGO_0160 encodes ZnuA, which chaperones Zn from the periplasm and delivers it to ZnuB. A simplified image of the *znuCBA* gene products is depicted. The gene products of the NGO_0170, NGO_0169, and NGO_0168 have been implicated in both Zn and manganese (Mn) binding and transport. Thus, *znuCBA* is also referred to as *mntABC* in reference to Mn transport. *znuCBA* and *mntABC* are two different names to identify the same genetic sequence indicated by the locus numbers NGO_0170, NGO_0169, and NGO_0168. The *znuCBA* operon including the intergenic regions in *N. gonorrhoeae* is 92% identical to that in *N. meningitidis* (Accession number AE002098.2), in which *znuCBA* is also responsible for Zn and Mn import.

*N. gonorrhoeae* express a zinc import system encoded by *znuCBA* (NGO_0170-_0168) (accession number NC_002946) where the gene products, ZnuC, ZnuB, and ZnuA are the ATPase, permease, and PBP, respectively (**Figure 2** and **Table 1**). ZnuCBA transports zinc through the periplasm and across the cytoplasmic membrane. A *znuA* mutant was growth deficient in the presence of all supplemental metals (i.e. Mg^2+^, Mn^2+^, Cu^2+^, Ni^2+^, Fe^2+^, Fe^3+^, Ca^2+^, and Cd^2+^) except Zn^2+^, demonstrating the specificity of this importer for zinc, over other metals, under these conditions (Chen and Morse, 2001). Growth only with supplemental zinc suggests that the gonococcus requires specific zinc import *via* ZnuA for cellular processes that cannot be completed with substituting metals under these conditions.

A manganese-specific outer membrane importer has not been identified in pathogenic *Neisseriae* despite the requirement for manganese, rather than zinc, cobalt, or magnesium, to resist oxidative killing (Tseng et al., 2001). However, the ability of TdfH to bind to manganese-loaded calprotectin suggests the possibility for highly specific manganese import across the outer membrane in the pathogenic *Neisseriae* (**Table 1**) (Bera et al., 2022*)*.

The *znuCBA* operon is also referred to as *mntABC* in the context of manganese transport through the periplasm and across the cytoplasmic membrane **(** **Table 1****)**. MntA, MntB, and MntC are the ATPase, permease, and PBP, respectively. *znuCBA* and *mntABC* are different names for the same operon. *znuCBA* was used to describe the operon when the gene products were demonstrated to be involved in zinc import; somewhat confusingly, *mntABC* was deployed as the term to describe the operon when the gene products were involved in manganese import (**Figure 2**). The gene locus is the same for both systems and presumably encodes the proteins required for both manganese and zinc import.

ZnuA (**Figure 2**), from Ngo strain FA1090 (accession number NC_002946) shares 96% amino acid identity with a zinc ABC transport PBP encoded by *N. meningitidis*. This high sequence similarity suggests that *N. meningitidis* also requires a PBP for zinc and manganese transport through the periplasm. A gonococcal *mntC* mutant imports 500-fold less manganese than the wild type, demonstrating a dual metal-binding capacity (Tseng et al., 2001). MntC binds manganese and zinc with nearly equal affinity (100 ± 8 nM for Mn^2+^ and 104 ± 5 nM for Zn^2+^), suggesting that the gonococcus occupies niches during infection that are limited in both metals (Chen and Morse, 2001; Lim et al., 2008). This import system may be required for growth and pathogenesis in *N. meningitidis* much like it is in *N. gonorrhoeae*.

Calmettes et al. showed that the meningococcal TdfJ homolog, ZnuD, crystalizes with zinc and cadmium (**Table 1**) at distinct binding sites in the absence of a chelator, suggesting the ability to bind both ions in their free form (Calmettes et al., 2015). Hecel et al. defined the metal binding specificity of the flexible loop responsible for ion capture by ZnuD (Hecel et al., 2019), noting that this flexible loop binds copper (**Figure 1**) with higher affinity and stability than it does zinc and that the loop undergoes a substantial conformational change upon copper binding (Hecel et al., 2019). The ability of ZnuD/TdfJ to bind copper suggests that this transporter may import copper in addition to zinc (**Figure 1**, **Table 1**).

A periplasmic copper chaperone has not yet been identified in the pathogenic *Neisseriae*. However, the ability of ZnuD to bind free copper suggests that copper could be imported through the outer membrane to the periplasm and may require a chaperone for delivery to the cytoplasmic membrane.

## Metal Availability Sensors Regulate Metal Import Genes

Metal-dependent regulation in bacteria is often accomplished by the ferric uptake regulator (Fur) -family proteins (Taylor-Robinson et al., 1990). Fur-family metalloregulators include Fur, PerR, Mur, Nur, Zur, and Irr, which are responsible for regulating iron uptake, peroxide stress sensing, manganese uptake, nickel uptake, zinc uptake, and heme-dependent iron uptake (Fillat, 2014). These Fur-family regulators are responsible for sensing metal availability within microenvironments inhabited by pathogens and subsequently coordinating a transcriptional response.

Pathogenic *Neisseriae* express two well-characterized metal dependent regulators: Fur and Zur (Zur has also previously been called PerR) (Wu et al., 2006; Pawlik et al., 2012; Jean, 2015; Jean et al., 2016). In *N. gonorrhoeae*, Zur is hypothesized to repress zinc and manganese import genes in the presence of these metals and to de-repress zinc and manganese import genes in the absence of these metals (**Table 1**) (Chen and Morse, 2001; Wu et al., 2006). Production of gonococcal proteins TdfJ and TdfH is zinc-repressed in a Zur-depending manner (Jean et al., 2016). In the context of infection at mucosal surfaces and in neutrophils, *N. gonorrhoeae* requires a zinc sensor, such as Zur, to mount a transcriptional response to calprotectin- and S100A7- mediated zinc limitation. Zur de-represses expression of high-affinity metal importers, including *znuCBA, tdfJ*, and *tdfH*, so that the gonococcus can effectively and efficiently import Zn. Meningococcal Zur specifically binds to the promoter of *znuD (P_znuD_)* in the presence of Zn^2+^, but not Ca^2+^, Co^2+^, Cu^2+^, Fe^2+^, Mg^2+^, Mn^2+^, or Ni^2+^. Zinc-dependent binding of Zur to *P_znuD_* is abrogated with the addition of a zinc-specific chelator, N,N,N′,N′-tetrakis (2-pyridinylmethyl)-1,2-ethanediamine (TPEN) (Pawlik et al., 2012), suggesting that Zur is responsible for sensing zinc availability and coordinating a transcriptional response that allows for zinc acquisition. Microarray and RT-qPCR analyses also showed zinc-dependent regulation of 11 other genes, including *cbpA*, *znuCBA*, the high-affinity zinc ABC importer, and genes encoding multiple ribosomal proteins, nitrosative stress resistance proteins, and metabolic proteins (Pawlik et al., 2012; Stork et al., 2013).

Manganese-dependent regulation by gonococcal Zur (also referred to as PerR) was demonstrated by Tseng et al. and Wu et al. (**Table 1**) (Tseng et al., 2001; Seib et al., 2006; Wu et al., 2006). Wu et al. established that *znuCBA (mntABC)* is manganese-repressed in a Zur-dependent manner. Manganese has also been shown to upregulate many ribosomal proteins, pilus assembly proteins, adhesion proteins, outer membrane proteins, the multidrug efflux pump protein channel, MtrE, and many metabolic proteins (Wu et al., 2010). Interestingly, the iron-repressed transferrin-binding protein A (TbpA) and the transport protein ExbB were also manganese-repressed (Ronpirin et al., 2001; Wu et al., 2010). These data suggest that gonococcal Zur senses manganese limitation during infection where calprotectin and potentially other manganese-binding proteins sequester manganese.

Copper sensing and regulation in bacteria are often accomplished by CueR, which is absent from the gonococcal genome (accession number NC_002946; Arguello et al., 2013). The genome of *N. meningitidis* encodes a putative CueR regulator (accession number MBF1297094.1) that is 47.62% identical to that found in *E. coli* (accession number NP_415020). Although, it has not yet been empirically characterized as a copper-dependent regulator. Neisserial MisR (accession number WP_002214312.1), is homologous to CueR. MisR is the response regulator of theMisR-MisS two-component regulatory system and is known to be involved in cationic antimicrobial peptide resistance (Kandler et al., 2016). Interestingly, MisR is 36% identical and 58% similar to *Pseudomonas aeruginosa* CopR, which is involved in regulation of copper homeostasis (Novoa-Aponte et al., 2020).

Much work is needed to characterize the ability of pathogenic *Neisseriae* to sense and regulate copper, considering that ceruloplasmin is found in the serum, which is a meningococcal infection site (Osaki et al., 1966). Ceruloplasmin concentrations in the cerebrospinal fluid (0.8-2.2 µg/mL) are 100-500-fold lower than that in the serum (Irani, 2008). The gonococcus may also need to sense copper levels, considering the potential for copper to fluctuate following CTR1 protein expression within neutrophils.

## Transition Metals Are Required for Survival and Virulence

Bacteria utilize scarce metals for several mechanisms related to survival and virulence, such as resistance to reactive oxygen species (ROS) and reactive nitrogen species (RNS), metabolism, maintenance of cell structural integrity, and proper protein structure and function.

Zn contributes to the function of biosynthetic pathways and virulence in *N. gonorrhoeae* and *N. meningitidis* as a cofactor for enzymes, enabling survival and virulence. For example, in both species, biosynthesis of lipid A, a potent immune activator (Mandrell et al., 1988; Steimle et al., 2016), involves a putative zinc-dependent metalloamidase, UDP-3-O-(R-3- hydroxymyristoyl)-N-acetylglucosamine deacetylase (LpxC) (Barb and Zhou, 2008; Mochalkin et al., 2008; John et al., 2018). Lipid A anchors LOS into the bacterial membrane and can activate the immune system after its release from the bacterial cell wall (Steimle et al., 2016). Additionally, it can be directly recognized by host Lipid-A binding protein (LPB), which plays a role in sensing of pathogenic (Knapp et al., 2003) and commensal species (Steimle et al., 2016). Thus, LOS is a key virulence factor in *N. gonorrhoeae* and *N. meningitidis*. Similarly, the *N. meningitidis* protein, Ght, a zinc binding protein involved in LOS biogenesis, (Putker et al., 2014) is involved in LOS expression and outer membrane integrity (Putker et al., 2014). These observations implicate zinc in virulence and survival.

Manganese contributes to oxidative stress resistance in the pathogenic *Neisseriae* and thus contributes to survival during infection at highly oxidative sites (Tseng et al., 2001; Wu et al., 2006). Manganese in bacteria cycles between the Mn^2+^ and Mn^3+^ states during MnSOD processing of reactive oxygen species (Abreu and Cabelli, 2010). Interestingly, the pathogenic *Neisseria* do not express a MnSOD and instead use manganese directly as an ROS quencher (Seib et al., 2006). Wu et al. demonstrated a role for gonococcal *mntC* in the oxidative stress response under anaerobic rather than aerobic conditions (Wu et al., 2009). The vagina and cervix are normally oxygen-depleted, making anaerobic gonococcal growth conditions highly relevant (Hill et al., 2005). *In vitro*, growth of an *mntC* mutant under anaerobic conditions was inhibited by paraquat, an intracellular inducer of ROS, to an extent similar to that of the wild type (Wu et al., 2009). However, the *mntC* mutant was less competitive than the wild type during *in vivo* infection, which is characterized by both anaerobic and highly oxidative conditions (Wu et al., 2009). Reduced competition by the *mntC* mutant under anaerobic and oxidative conditions suggests that manganese is critical to gonococcal growth within the cervical niche. In contrast to the gonococcus, growth of the meningococcus in the presence of manganese does not enhance oxidative stress resistance (Seib et al., 2004).

*N. meningitidis* is able to grow on manganese concentrations 50-100 times higher (>100 mmol/L) than *N. gonorrhoeae*, which suggests that manganese homeostasis differs between these species (Tseng et al., 2001; Seib et al., 2004). Despite the non-restorative effect of manganese during oxidative conditions in the meningococcus, manganese is a vital cofactor in biosynthetic pathways within the bacterium. Meningococcal sialic acid synthase, NeuB, was shown to crystalize best with the addition of manganese, suggesting that this enzyme also requires a manganese cofactor (Tseng et al., 2001; Gunawan et al., 2005). NeuB is involved in sialylated capsule formation (Gunawan et al., 2005), and the sialylated surface of *N. meningitidis* has been shown to protect the bacterium from complement deposition (van Emmerik et al., 1994) through molecular mimicry of host cell surface proteins (Mandrell et al., 1988). Due to the potential involvement of manganese in NeuB activity, and consequently capsule formation, the metal may play a role in protection from host complement deposition and thus in immune evasion. This mechanism of innate immune evasion is particularly useful to the pathogen during infection of the vasculature, a niche that is complement is enriched.

In the pathogenic *Neisseria*, copper plays a role in resistance to extracellular RNS (Mellies et al., 1997; Boulanger and Murphy, 2002; Seib et al., 2006). *N. gonorrhoeae* (Gotschlich and Seiff, 1987) and *N. meningitidis* (Woods et al., 1989) possess a surface-exposed lipid-modified azurin (Desrosiers et al. 2010), which is a putative electron donor to peroxidases (Seib et al., 2006). This biological function is particularly relevant to macrophage infection because they are known to increase the expression of nitric oxide synthase (NOS) upon stimulation with LOS (Blondiau et al., 1994; Iovine et al., 2008). *N. gonorrhoeae* can survive in the harsh environment of macrophage phagosomes, potentially through a mechanism involving copper-bound Laz (Château and Seifert, 2016; Quillin and Seifert, 2018). Interestingly, the gonococcal genome encodes a putative peptidase with a PepSy domain (Accession number WP_003702955.1). This peptidase is 34% identical and 51% similar to that produced by *P. aeruginosa* (NCBI Reference Sequence: NP_252478.1). The peptidase in *P. aeruginosa* has been shown to be copper-repressed (Quintana et al., 2017). A similar putative peptidase is predicted to be produced by the meningococcus (Accession number WP_079889394.1) and is 36% identical and 50% similar to that from *P. aeruginosa*. The role of copper in the regulation and function of this peptidase in *N. gonorrhoeae* and *N. meningitidis* is a potential focus of future investigation.

## The Response to Metal Overload Requires Sensing and Export of Intoxicating Metals

The host applies metal intoxication strategies to limit bacterial growth and survival. Metal intoxication is the process by which the host floods the bacterial nutrient supply with metals. The consequences of metal intoxication for bacteria include electron transport chain (ETC) inhibition, protein mismetallation, and ROS and RNS accumulation (Chandrangsu et al., 2017). To limit these consequences, bacteria utilize mechanisms that store or export excess metal and repress metal import systems (Chandrangsu et al., 2017). Metal toxicity in *N. gonorrhoeae* has also been shown in reference to Mn^2+^, Co^2+^, Ni^2+^, and Zn^2+^ (Odugbemi et al., 1978; Veyrier et al., 2011). However, specific responses to overload of each metal remain poorly characterized.

While macrophages have not been shown to exert metal intoxication upon the pathogenic *Neisseria* species, these immune cells have demonstrated the ability to increase the concentration of zinc in the cytosol and the phagocytic vacuole *via* the SLC39A transporters (Begum et al., 2002; Stafford et al., 2013; Chandrangsu et al., 2017) suggesting that host-induced zinc toxicity may be relevant to pathogenic *Neisseria* infection. Macrophages have been shown to increase phagosomal zinc concentrations to apply metal stress on invading *Mycobacterium* species (Wagner et al., 2005; Lefrançois et al., 2019). In *Streptococcus pneumoniae*, excess zinc competes with manganese for binding to pneumococcal surface antigen A (PsaA), resulting in reduced manganese uptake, reduced oxidative stress resistance, and reduced resistance to PMN killing (McDevitt et al., 2011). In *E. coli*, high levels of manganese correlate with reduced levels of Fe^2+^, iron-containing enzymes in the ETC and TCA cycle (iron-sulfur clusters and heme-containing enzymes), and consequently, reduced levels of NADH and ATP (Kaur et al., 2017).

Although not empirically tested in the gonococcus, manganese intoxication has been tested and shown to be relevant to meningococcal growth. Excess manganese in *N. meningitidis* results in protein mismetalation and subsequent dysregulation of Fur-regulated genes (**Table 1**) (Veyrier et al., 2011). Under excess manganese conditions, the meningococcus expresses *mntX*, the gene encoding a manganese export protein, which is critical to survival under high manganese conditions. MntX contains predicted transmembrane domains suggesting that this protein transports manganese from the cytoplasm to the periplasm (Veyrier et al., 2011). The *mntX* mutant exhibited a reduced ability to survive in the blood of infected mice relative to the wild-type strain (Veyrier et al., 2011). Additionally, the *mntX* mutant showed reduced resistance to human serum (Veyrier et al., 2011). Taken together, these data suggest that the meningococcus senses high manganese during septicemic infection and responds by expressing *mntX*. It is also feasible that the meningococcus requires a manganese exporter during infection in the cerebrospinal fluid. The blood and cerebrospinal fluid are body sites that are manganese-enriched and may be a hostile environment for a pathogen lacking a Mn exporter. Metal toxicity in *N. meningitidis* has also been shown in reference to Cu^2+^, Co^2+^, Ni^2+^, and Zn^2+^ (Odugbemi et al., 1978). Conversely, *mntX* is frameshifted in 66% of sequenced *N. gonorrhoeae* strains. A *N. gonorrhoeae* strain, which was sensitive to manganese in this way, could be rescued through complementation with meningococcal *mntX* (Veyrier et al., 2011). Expression of *mntX* in certain gonococcal strains may enable dissemination to the blood and meninges.

Copper intoxication is used by the host to limit bacterial infection. For example, copper influx into the phagosomal compartment of macrophages results in increased killing of an *E. coli* strain deficient in a copper efflux protein, CopA, relative to the wild type (White et al., 2009). The gonococcus also produces a copper efflux protein, CopA, in the cytoplasmic membrane (Djoko et al., 2012). CopA likely transports copper from the cytoplasm to the periplasm. A *copA* mutant exhibited higher concentrations of internal copper and was growth impaired under high copper conditions (Djoko et al., 2012). Following copper supplementation, the *copA* mutant was limited in its ability to associate with and invade primary human cervical epithelial cells; it was also less resistant to killing by nitrite and *S*-nitrosoglutathione (GSNO), a nitric oxide generator (Djoko et al., 2012). This data suggest *N. gonorrhoeae* experiences copper overload within cervical epithelial cells. Djoko and McEwan showed that high levels of copper increase gonococcal sensitivity to the RNS generator, sodium nitrite and suggested that copper-dependent inactivation of hemoproteins involved in intracellular RNS detoxification results in RNS-dependent killing of *N. gonorrhoeae* (Djoko and McEwan, 2013).

While the concept of nutritional immunity has been thoroughly studied, the concept of metal intoxication requires further exploration. Expanded application of this concept to pathogenic *Neisseriae* infection will broaden our understanding of metal homeostasis and its role in pathogenesis.

## Next Steps - Comprehensive Characterization of Nutritional Immunity and Metal Intoxication Requires Insight Into Integrated Metal Homeostasis

Pathogens possess mechanisms of defense against metal starvation and metal intoxication exerted by the host, as these metals play integral roles in metabolism, maintenance of cell structural integrity, and ROS and RNS resistance. Metal involvement in these processes is often studied in isolation, meaning only one metal (i.e., zinc, manganese, or copper) is considered at a time. However, it is improbable that pathogens encounter a single type of metal depletion or stress during infection of a host whose metal allocations and concentrations are heterogeneous.

One of the most thoroughly investigated examples of metal interplay is that between manganese and zinc in *S. pneumoniae.* Cell-associated manganese in wild-type *S. pneumoniae* is substantially reduced in the presence of excess zinc, and zinc depletion of cellular manganese could be restored by the addition of excess manganese (Jacobsen et al., 2011). Under high zinc conditions, zinc competes with and inhibits manganese binding to the manganese importer, PsaA, resulting in manganese starvation and zinc toxicity (Jacobsen et al., 2011; Eijkelkamp et al., 2014). Zinc-induced manganese starvation leads to increased sensitivity to oxidative stress (Eijkelkamp et al., 2014). Another example of metal interplay has been investigated in *S.enterica* Serovar Typhimurium. In this bacterium, *mntH* encodes a manganese importer that is iron-repressed in a Fur-dependent manner and manganese-repressed in a manganese transport repressor (MntR) -dependent manner (Kehres et al., 2002a). Kehres et al. hypothesized that co-regulation of *mntH* maintains an equilibrated Mn^2+^/Fe^2+^ ratio in *Salmonella* (Kehres et al., 2002a). The genomes of *N. gonorrhoeae* and *N. meningitidis* encode a MntH homolog; however, the functions of this Fe^2+^/Mn^2+^ symporter in manganese or iron import have yet to be tested in the *Neisseriae*. *Helicobacter pylori* expresses a metal efflux system, CznABC, which interacts with cadmium, zinc, and nickel and confers resistance to intoxicating levels of all three metals (Stähler et al., 2006). *Acinetobacter baumannii*, when grown in the presence of calprotectin that is able to simultaneously chelate zinc and manganese, exhibits reduced intracellular manganese and zinc but increased iron levels (Hood et al., 2012). In this case, calprotectin treatment not only resulted in altered zinc, manganese, and iron homeostasis but also in reduced growth (Hood et al., 2012). In *Klebsiella pneumoniae*, the zinc efflux protein, ZntA, is responsible for exporting zinc from the cytoplasm (Maunders Eve et al., 2022). The *zntA* mutant was shown to accumulate more manganese in addition to zinc and less iron than the wild type when subject to high zinc conditions, demonstrating integrated zinc, manganese, and iron homeostasis in wild type *K. pneumoniae* (Maunders Eve et al., 2022).

Pathogenic *Neisseriae* sense metal concentrations in the environment and respond by altering the expression of metal import or export systems and allocating these metals to metabolic and biosynthetic processes to allow for survival and virulence. *N. gonorrhoeae* and *N. meningitidis* are similar pathogens, which respond similarly to metal limitation and overload within the same host, despite causing different physiological symptoms. It is unlikely that the pathogenic *Neisseria* experience zinc, manganese, and copper starvation or intoxication in isolation from other metals. Interaction with multiple metals by neisserial proteins suggests the need for complex and integrated metal homeostasis **(** **Table 1****)**. This is evidenced by the ZnuCBA manganese import system in *N. gonorrhoeae*, which is manganese-regulated in a Zur-dependent manner but is also responsible for zinc import (**Table 1**) (Chen and Morse, 2001; Tseng et al., 2001; Wu et al., 2006). More work needs to be done to characterize Zur metal sensing when zinc and manganese are present together. Gonococcal TdfJ is zinc-repressed in a Zur-dependent manner and is iron-induced (Jean et al., 2016). The zinc to iron ratio required for optimal TdfJ expression in the presence of S100A7 should be addressed in future studies. Gonococcal TbpA (**Table 1**) is both iron- (Ronpirin et al., 2001; Vélez Acevedo et al., 2014) and manganese-repressed (Wu et al., 2010). TbpA is an iron-repressed (Ronpirin et al., 2001; Vélez Acevedo et al., 2014) TonB-dependent transporter that pirates iron from human transferrin and transports it across the outer membrane (Noto and Cornelissen, 2008; Cash et al., 2015). Considering that TbpA is also manganese-repressed and that transferrin can bind manganese in a manner similar to iron, it would be interesting to determine whether TbpA can pirate manganese from manganese-loaded transferrin. Additionally, studies regarding the application of this potential manganese transport system to a host niche would be informative. The manganese export protein, MntX, in *N. meningitidis* is required for survival under high manganese and low iron conditions, and the absence of the gene encoding this system results in mis-regulation of iron-regulated genes (**Table 1**) (Veyrier et al., 2011). It would be informative to discern the exact manganese to iron ratio required for optimal *mntX* expression and consequent serum resistance in *N. meningitidis.* In-frame MntX is only present in a subset of gonococcal strains (Veyrier et al., 2011).

The evolution of complex metal regulatory and import mechanisms suggests that the pathogenic *Neisseriae* possess a need for multifactorial metal homeostasis. Instances of overlap in different metal-related processes imply that the pathogenic *Neisseriae* may specifically require the integration of manganese and iron homeostasis and zinc and copper homeostasis. The exact mechanisms of integrated metal homeostasis and the host conditions under which they are relevant have not yet been fully deciphered. Further investigation into the complex metal environment sensed by the bacteria in the host could broaden our understanding of mixed metal homeostasis and the neisserial response to nutritional immunity and metal intoxication.

## Author Contributions

AHB completed the literature review and manuscript drafting and editing based on JLS, KSL, and CNC comments. JLS reviewed and proofread the manuscript multiples times before review by KLS. CNC then reviewed and proofread the manuscript and acquired funding. All authors contributed to the article and approved the submitted version.

## Funding

This work was funded by the National Institute of Allergy and Infectious Diseases, award numbers U19AI144182, R01AI127793, and R01AI125421 to CC. The funder had no role in data collection, synthesis, analysis, interpretation, or management of the data presented in this review. The funder had no role in review generation and revision, or in the decision to submit this review for publication.

## Conflict of Interest

The authors declare that the research was conducted in the absence of any commercial or financial relationships that could be construed as a potential conflict of interest.

## Publisher’s Note

All claims expressed in this article are solely those of the authors and do not necessarily represent those of their affiliated organizations, or those of the publisher, the editors and the reviewers. Any product that may be evaluated in this article, or claim that may be made by its manufacturer, is not guaranteed or endorsed by the publisher.
